# Deep Learning-Based Real-Time Organ Localization and Transit Time Estimation in Wireless Capsule Endoscopy

**DOI:** 10.3390/biomedicines12081704

**Published:** 2024-07-31

**Authors:** Seung-Joo Nam, Gwiseong Moon, Jung-Hwan Park, Yoon Kim, Yun Jeong Lim, Hyun-Soo Choi

**Affiliations:** 1 Division of Gastroenterology and Hepatology, Department of Internal Medicine, Kangwon National University School of Medicine, Chuncheon 24341, Republic of Korea; 2 Ziovision Co., Ltd., Chuncheon 24341, Republic of Korea; 3 Department of Computer Science and Engineering, College of IT, Kangwon National University, Chuncheon 24341, Republic of Korea; 4 Division of Gastroenterology, Department of Internal Medicine, Dongguk University Ilsan Hospital, Dongguk University College of Medicine, 27 Dongguk-ro, Ilsandong-gu, Goyang 10326, Republic of Korea; 5 Department of Computer Science and Engineering, Seoul National University of Science and Technology, 232, Gongneung-ro, Nowon-gu, Seoul 01811, Republic of Korea

**Keywords:** wireless capsule endoscopy, deep learning, gastrointestinal transit

## Abstract

Background: Wireless capsule endoscopy (WCE) has significantly advanced the diagnosis of gastrointestinal (GI) diseases by allowing for the non-invasive visualization of the entire small intestine. However, machine learning-based methods for organ classification in WCE often rely on color information, leading to decreased performance when obstacles such as food debris are present. This study proposes a novel model that integrates convolutional neural networks (CNNs) and long short-term memory (LSTM) networks to analyze multiple frames and incorporate temporal information, ensuring that it performs well even when visual information is limited. Methods: We collected data from 126 patients using PillCam™ SB3 (Medtronic, Minneapolis, MN, USA), which comprised 2,395,932 images. Our deep learning model was trained to identify organs (stomach, small intestine, and colon) using data from 44 training and 10 validation cases. We applied calibration using a Gaussian filter to enhance the accuracy of detecting organ boundaries. Additionally, we estimated the transit time of the capsule in the gastric and small intestine regions using a combination of a convolutional neural network (CNN) and a long short-term memory (LSTM) designed to be aware of the sequence information of continuous videos. Finally, we evaluated the model’s performance using WCE videos from 72 patients. Results: Our model demonstrated high performance in organ classification, achieving an accuracy, sensitivity, and specificity of over 95% for each organ (stomach, small intestine, and colon), with an overall accuracy and F1-score of 97.1%. The Matthews Correlation Coefficient (MCC) and Geometric Mean (G-mean) were used to evaluate the model’s performance on imbalanced datasets, achieving MCC values of 0.93 for the stomach, 0.91 for the small intestine, and 0.94 for the colon, and G-mean values of 0.96 for the stomach, 0.95 for the small intestine, and 0.97 for the colon. Regarding the estimation of gastric and small intestine transit times, the mean time differences between the model predictions and ground truth were 4.3 ± 9.7 min for the stomach and 24.7 ± 33.8 min for the small intestine. Notably, the model’s predictions for gastric transit times were within 15 min of the ground truth for 95.8% of the test dataset (69 out of 72 cases). The proposed model shows overall superior performance compared to a model using only CNN. Conclusions: The combination of CNN and LSTM proves to be both accurate and clinically effective for organ classification and transit time estimation in WCE. Our model’s ability to integrate temporal information allows it to maintain high performance even in challenging conditions where color information alone is insufficient. Including MCC and G-mean metrics further validates the robustness of our approach in handling imbalanced datasets. These findings suggest that the proposed method can significantly improve the diagnostic accuracy and efficiency of WCE, making it a valuable tool in clinical practice for diagnosing and managing GI diseases.

## 1. Background

Currently, deep learning-based technologies are being actively applied in every field of medicine. In gastroenterology, one of the most common clinical applications of artificial intelligence is lesion detection and characterization during endoscopic examination [1,2]. For wireless capsule endoscopy (WCE), various algorithms have been developed focusing on lesion detection, aiming to reduce clinicians’ reading time and workload [3,4,5,6,7,8,9,10]. However, there are limited studies on the localization of WCE (i.e., systems that inform the examiner where the capsule is located, such as in the esophagus, stomach, small intestine, or colon) [11,12]. The overlooked function of capsule localization can be valuable in various clinical situations. One of the major limitations of WCE is the frequent incomplete examination rate, which is reported to be 20–30% [13,14]. Delayed gastric transit time has been a leading cause of incomplete studies, and several clinical guidelines recommend additional intervention, such as the administration of prokinetic agents or endoscopic capsule delivery into the duodenum, in the case of gastric capsule retention for more than one hour [14,15]. Plain radiography or real-time viewer has been recommended for the detection of gastric retention, but this can be another burden to the clinician and makes WCE difficult in the outpatient setting [16]. Capsule localization, especially, informing the entrance into the small intestine with the aid of artificial intelligence can address these issues. Also, correct localization can make new capsule devices such as colon capsule or new capsule methods such as the diving method easily applicable in a clinical setting [17,18].

Previous studies have mainly relied on creating feature vectors by combining color, texture, and motion information, or classification models that make decisions based on a single image [11,19,20,21,22,23]. In such cases, the performance significantly decreases when there is a lot of noise or the presence of food debris or fecal material. Recently, deep learning algorithms have been utilized to overcome these limitations. While some studies have explored the use of convolutional neural networks (CNNs) and temporal filtering to localize regions of interest, these approaches did not consider the sequence information of the continuous videos [12].

To address this limitation, we combined the strengths of both CNN and long short-term memory (LSTM) models, enabling our algorithm to learn the sequence information of the video. Our model maintains robust performance by combining temporal data with color information even when visual information is insufficient. Furthermore, we applied calibration techniques to our model to detect each organ’s boundary with greater accuracy. This approach enhances the reliability of organ classification and localization in capsule endoscopy, ensuring more stable and accurate results in clinical practice. We evaluated the performance of our algorithm on WCE videos and obtained promising results, demonstrating the potential of our approach for improving the accuracy of transit time detection in the gastrointestinal tract.

## 2. Methods

**Figure 1:**
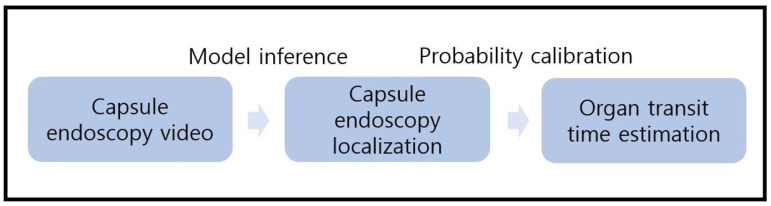
Technical flowchart that shows the overall system development process.

The technical flowchart of the development of landmark detection system for the stomach, small intestine, and colon is shown in Figure 1. First, we built a classification model for organ localization. Subsequently, we implemented a calibration method to efficiently predict the time points at which organ transitions occur in the capsule video. The study protocol was reviewed and approved by the Dongguk University Ilsan Hospital (Ilsan, Republic of Korea) Institutional Review Boards (IRBs) in accordance with the ethical principles outlined in the Declaration of Helsinki. The IRB approval number for this study is 2022-01-032. Informed consent was waived due to the study’s retrospective nature and the use of de-identified data by the Dongguk University Ilsan Hospital Institutional Review Boards.

### 2.1. Dataset

**Figure 2:**
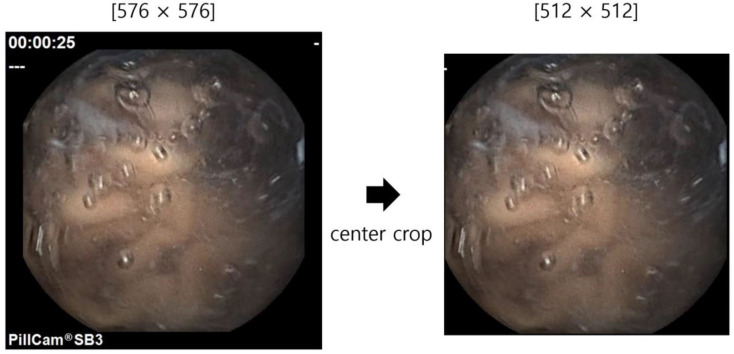
Image preprocessing using center crop.

We analyzed the data of the PillCam™ SB3 (Medtronic, Minneapolis, MN, USA) capsule endoscopy system from two university-affiliated hospitals (Kangwon National University Hospital (Chuncheon, Republic of Korea) and Dongguk University Ilsan Hospital) between 2018 and 2021. Table 1 shows the number and classification of the main pathologic findings in the dataset, which comprises a total of 126 patients. We extracted the video using the Pillcam™ Reader Software v9.0. Each video had a duration ranging from 8 to 13 h and a frame rate between 2 and 6 frames per second. Converting capsule video images into frames results in a large amount of training data, with over 300,000 images for each video. Thus, we extracted (downsampled) one frame from every five frames, resulting in data comprising 2,395,932 images from the dataset. The images obtained from WCE are labeled with four locations, namely, the esophagus, stomach, small intestine, and colon. For clinical and practical reasons, as explained in the Experiments section (4.2), we defined only three locations in this study: the stomach, small intestine, and colon. Therefore, the total number of images excluding the esophagus was 2,392,462. Subsequently, training, validation, and test data were separated, as shown in Table 2. In order to ensure the even distribution of all types of lesions, we set the training/validation to test ratio to 2:3 for each category, except for the normal category, for which we set the ratio to 1:1 due to the sufficient test data. The number of images for each label is shown in Table 3. All images were colored and had a resolution of 576 × 576. However, the endoscopic product name and examination time were shown on each image, making it challenging to use as training data. Therefore, we cropped 512 × 512 around the center, as shown in Figure 2. We loaded the data in sequences of 32 images for the CNN including sequential models, resulting in one sequence having the following format [L,C, H, W]=[32, 3, 512, 512], where *L* represents sequence length in video, *C* represents the number of color channels, and *H* and *W* represent the height and width of the images, respectively.

### 2.2. Convolutional LSTM Considering Sequence Awareness

**Figure 3:**
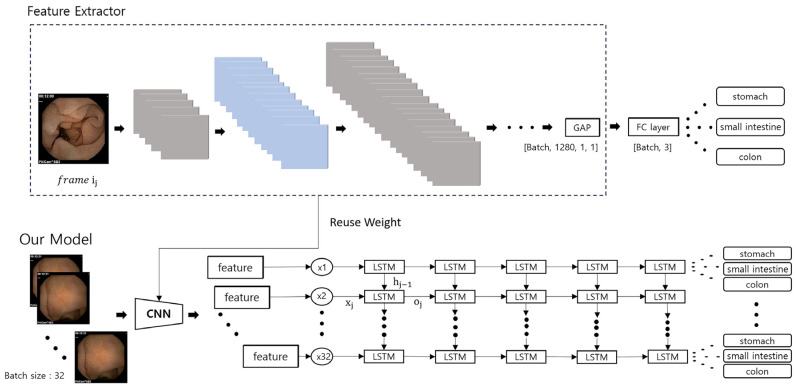
Diagram of the proposed model, which combines a conventional neural network (CNN) and a long short-term memory (LSTM). The CNN is used for feature extraction and the LSTM is used to learn sequence awareness.

In this study, we propose a model that incorporates LSTM to learn sequential order awareness in videos. Our model consists of a feature extraction network constructed with a CNN, which is then followed by an LSTM layer, as shown in Figure 3. We chose to use a pre-trained EfficientNet with noisy student training for the feature extractor, which has fewer trainable parameters and demonstrated relatively superior performance [24] in our experiments. The core building block of EfficientNet is the MBConv (Mobile Inverted Bottleneck Convolution) layer, which includes depthwise separable convolutions and squeeze-and-excitation optimization. This design reduces computational cost while maintaining high performance. EfficientNet has several variants (B0 to B7), each scaled to different sizes. We selected EfficientNet-B0 for our model due to its balance of performance and computational efficiency.

The CNN extracts 1280-dimensional feature vectors from each frame, which are then processed sequentially by the LSTM to capture temporal dependencies. This architecture effectively combines the spatial feature extraction capabilities of CNNs with the temporal sequence learning abilities of LSTMs. In our model, the LSTM layer consists of five layers with LSTM units set to 64, allowing the model to learn the sequential patterns from 32 images of feature vectors obtained from the CNN. Each LSTM unit comprises a cell state and three gates. The cell state is responsible for keeping track of long-term dependencies, while the gates control the flow of information into and out of the cell state. The input gate controls how much new information from the current input should be added to the cell state. The forget gate decides what portion of the information in the cell state should be discarded. The output gate determines the amount of information from the cell state that should be passed to the next hidden state. These gates work together to retain important information over long sequences. In contrast, irrelevant information is filtered out, enabling the LSTM to capture long-term dependencies in the data. This mechanism allows our model to effectively process and understand the temporal context of the WCE video frames.

Let $i_{j}$ denote a j  input frame with the ground-truth labels stomach, small intestine, and colon. The compressed feature x with the classification labels is computed as:

(1) $$x_{j}=f_{GAP}(f_{Backbone}(i_{j};\theta_{Backbone}))\in R^{1280},$$

where $f_{GAP}$(·) represents the operation of global average pooling, $f_{Backbone}(\cdot )$ denotes the feature extraction from the backbone structure with parameter $\theta_{Backbone}$. And then, the sequence of frames in the video clip is represented by $X=[x_{1,},x_{2},\ldots x_{T}]$ where T is time as 32. We use $f_{j}$ to denote the representative image feature of each single frame $x_{j}$. The image features $f_{j}$ of the video clips are sequentially put into an LSTM network, which is denoted by $U_{\theta }$ with parameters θ. With the input $x_{j}$ and the previous hidden state $h_{j-1}$, the LSTM calculates the output $o_{j}$ and the updated hidden state $h_{j}$ as $o_{j}=h_{j}$= $U_{\theta }(x_{j}$, $h_{j-1}$). Lastly, the prediction of frame $x_{j}$ is generated by feeding the output $o_{j}$ into the softmax function. 

The way we trained a model that combines CNN and LSTM as shown in Figure 3 follows the order below. First, after conducting supervised learning through CNN, the classifier part is removed. The LSTM is then learned by reusing the weights of the CNNs whose learning has ended. It should be noted that the first loss function $L_{cnn}$ is the cross-entropy which is defined as

(2) $$L_{cnn}\left(i_{j},y_{j}\right)=-y_{j}\times \log f(i_{j}),$$

where f (·) represents the operation of the CNN, and $y_{j}$ denotes the ground truth of frame $i_{j}$. And the second loss function $L_{LSTM}$ is the cross-entropy which is defined as

(3) $$L_{LSTM}\left(X,Y\right)=\sum_{j=1}^{T}-y_{j}\times \log o_{j}$$

where Y denotes the ground truth of X vector. And $L_{LSTM}$ calculation consists of each loss sum of frame $x_{j}$ within T.

Our proposed model enables the learning of temporal dependencies within the videos and can improve the accuracy of tasks such as transit time detection in wireless capsule endoscopy examinations.

### 2.3. Probability Calibration for Detecting Organ Boundaries

**Figure 4:**
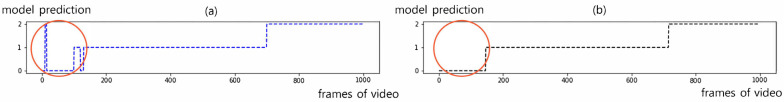
The effect of probability calibration. One case before the application of the Gaussian filter (**a**), and the same case after the application of the Gaussian filter (**b**). The *X*-axis represents the frames of the video. The *Y*-axis represents the model’s prediction, which classifies the location into the stomach (0), small intestine (1), and colon (2). The red circle denotes the effect of the Gaussian filter.

**Figure 5:**
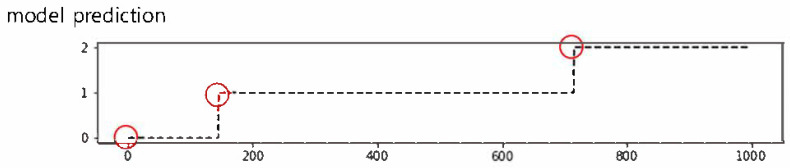
Example of anatomical landmark detection. Red circles indicate the transition points.

Probability calibration is needed for the results from the model because bouncing values occur, as shown in Figure 4a. We applied the Gaussian Filter Method to solve this problem. This function smooths the model’s results based on the Gaussian distribution. The result of windowing with the Gaussian filter is shown in Figure 4b. We performed an organ localization with the result from this correction. The organ boundaries we predict are determined as the part where the location changes, as shown in Figure 5.

### 2.4. Experiments and Evaluation

(1) Implementation details

Our model was trained for 20 epochs, with early stopping applied. We used the Sharpness-Aware Minimization optimizer with a batch size of 32 for training the CNN, and the Adam optimizer with a batch size of 1 for training the LSTM [25]. The training environment was a GPU RTX 3060 (Nvidia, Santa Clara, CA, USA), and the Pytorch deep learning framework was used. Hyper-parameters for probability calibration were determined using a validation dataset, and the post-processing method that provided the best results was a Gaussian filter with a size of 128, as shown in Appendix A. In clinical practice, landmarks should be predicted by analyzing real-time video images, so a half-Gaussian filter was used for calibration. We also skipped the model inference result for the first 512 frames to account for any potential noise or artifact at the start of the video. We applied brightness, contrast, saturation, and hue adjustments to the data, with a sampling probability of 0.8 among the batch data. Grayscale and horizontal flip transformations were used with a sampling probability of 0.5. We also randomly rotated images by 0, 90, 180, 270, or 360 degrees among the batch data. Additionally, we used Mix-up for generalization, which combined two images with a beta distribution of two different images [26].

(2) Evaluation strategies

To evaluate the performance of the model, we used standard computer vision metrics such as accuracy, F1-score, sensitivity, and specificity for each class. In addition, we assessed the model’s clinical feasibility by analyzing the organ transit time. Each transition point from the esophagus to the stomach, the stomach to the small intestine, and the small intestine to the colon were manually marked by the clinician. Based on these ground truths, we calculated the time difference between the model-predicted and manually marked organ transition points to evaluate the clinical feasibility of the developed model. To cover the various clinical situations, the test dataset included normal and several pathological cases, which are presented in Table 1 and Table 2 (specifically, 72 test datasets consisting of 24 normal, 12 bleeding, 18 inflammatory, 9 vascular lesion, and 9 polypoid lesion cases). Wilcoxon signed-rank test was performed for the statistical analysis on the difference in prediction errors of organ transition points between the stomach and small intestine, and the small intestine and colon. Kruskal–Wallis test was performed for the statistical analysis on the differences in prediction errors of organ transition points among different small intestine pathologies.

The clinical usefulness of checking the transition point between the esophagus and stomach is limited because, in almost all cases, the capsule’s entrance into the stomach is confirmed at the time when the capsule is swallowed, with the aid of a real-time viewer provided by the Pillcam™ recorder. Furthermore, the number of esophageal images acquired by the capsule endoscope is very limited (usually up to 10–100 images/patient), making it difficult to develop an accurate model. Therefore, we excluded esophageal images from the dataset and focused on predicting the transition time between the stomach and small intestine, which has the most potential for clinical applications.

## 3. Results

### 3.1. Performance Enhancement via Sequence-Aware Learning

We compared our proposed model, which incorporates LSTM for sequence awareness, to a baseline model that does not account for sequential order. The sequence-aware model achieved higher accuracy and F1-score, both at 0.971, compared to the baseline model, which had an accuracy of 0.961 and an F1-score of 0.960. The performance for each class is detailed in Table 4. Despite the small intestinal images comprising 70–80% of the total dataset due to the characteristics of the WCE, our proposed model demonstrated high specificity for the small intestine, particularly with sequence awareness. 

The Matthews Correlation Coefficient (MCC) is useful for evaluating models with imbalanced class distributions, as it provides a balanced measure that can handle such scenarios better than simple accuracy. The Geometric Mean (G-mean) is the square root of the product of sensitivity and specificity, balancing these two metrics. G-mean is also valuable for imbalanced datasets, as it ensures that the model performs well across all classes rather than being biased towards the majority class. These metrics demonstrate that our proposed sequence-aware model improves upon the baseline model in terms of accuracy and F1-score and shows superior performance in MCC and G-mean, indicating a better handling of imbalanced class distributions.

### 3.2. Probability Calibration

**Figure 6:**
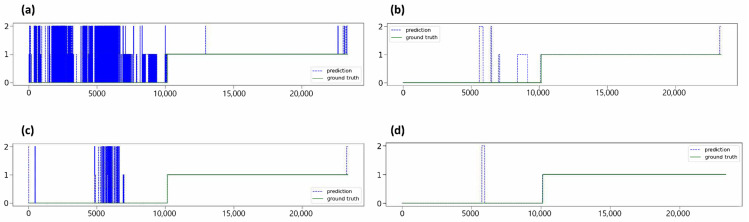
Comparison of the model’s prediction in terms of sequence awareness and probability calibration. The top two figures show organ prediction by the model without sequence awareness (**a**,**b**), and the bottom two figures are from the sequence-aware model (**c**,**d**). The two figures on the left represent the results before probability calibration (Gaussian filter) was applied (**a**,**c**), and the two pictures on the right represent the results after the application of the Gaussian filter (**b**,**d**). The *X*-axis represents the frames of the video. The *Y*-axis represents the model’s prediction, which classifies the location into the stomach (0), small intestine (1), and colon (2).

We evaluated both our proposed model and the baseline model using the test dataset videos, comparing prediction before and after applying probability calibration. The result indicated that, after applying probability calibration, the model had a lower frequency of false prediction and showed an improved detection of organ boundaries (Figure 6).

### 3.3. Prediction of the Organ Transition Points

**Figure 7:**
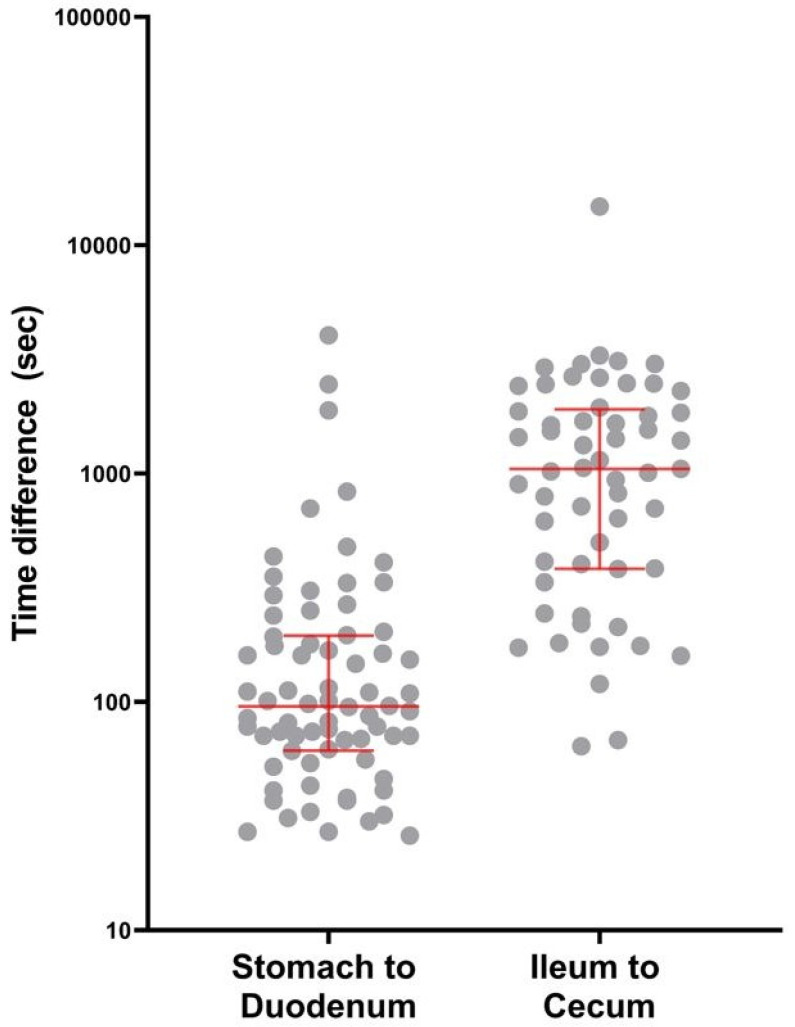
Time difference between the clinician’s decision and the model’s prediction. The red line represents the median value with 0.25 and 0.75 quartiles.

**Figure 8:**
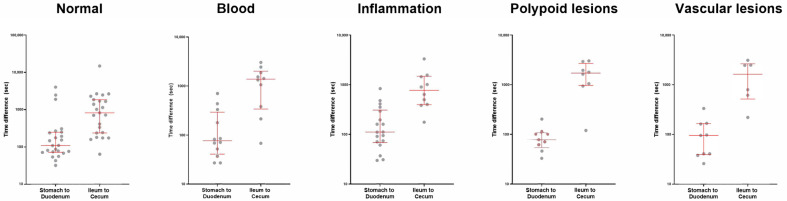
Time difference between the clinician’s decision and the model’s prediction across different pathologies of the small intestine. The red line represents the median value with 0.25 and 0.75 quartiles.

We calculated the time difference between the clinician’s manually defined transition point and the model-predicted point between the stomach and duodenum or the ileum and colon. The mean difference for the stomach to duodenum transition was 4.3 min, with a standard deviation of 9.7 min. The mean difference for ileum to colon transition was 24.7 min, with a standard deviation of 33.8 min. The time differences are larger for the ileum to colon transition than for the stomach to duodenum transition (*p*-value < 0.001). The overall distributions of the time difference between the clinician’s decision and the model’s prediction were presented in Figure 7. We also evaluated whether the small intestine pathologies such as bleeding, inflammation, polyps, or vascular lesions affected the model’s accuracy. The distributions of the time difference were similar across the different pathologies, indicating that these conditions did not significantly impact the accuracy (stomach to duodenum, *p*-value = 0.479; ileum to colon, *p*-value = 0.585; Figure 8).

Among the total test dataset of 72 cases, there were 14 cases with incomplete study (i.e., the capsule did not enter the colon). For 12 of these 14 incomplete cases, the model also predicted that the capsule had not passed into the colon until the last image. For the remaining two cases, the model predicted that the capsule had passed into the colon. Conversely, in 1 case among 58 cases with complete studies, the model could not predict the capsule’s entry into the colon even though the capsule had passed into the colon. For predicting the capsule’s entrance into the small intestine, there was no case that the model failed to predict it among the whole dataset of 72 cases.

## 4. Discussion

In this study, we developed a deep-learning model for real-time capsule localization based on the combination of CNN and LSTM. We evaluated the performance in both research basis (accuracy, F1-score, sensitivity, and specificity) and clinical aspects (time difference between the clinician’s and model’s predictions). The model showed good sensitivity, specificity, accuracy, and F1-score of over 95% in classifying images of the stomach, small intestine, and colon. Our model also showed good performance in predicting the capsule’s entrance into the small intestine, with a mean time difference of 258 s from the clinician’s manual annotation. In the 72 cases of the total test dataset, all the model’s predictions were within 15 min, showing clinical feasibility except 3 cases. Interestingly, these three cases had some unusual features. In the two cases showing a prediction delay of 30 and 40 min for each, the capsule stayed in the duodenal bulb for a long time (around 30 and 40 min) after the entrance into the duodenum, capturing multiple pylorus images (Appendix B, also known as the dark side of the pylorus) [27], and then passed into the distal duodenum and jejunum at the time point of the model’s prediction of small intestine entrance. We assume that multiple pylorus images from the duodenal side make our model infer that the capsule stayed in the stomach. Clinically, these situations are unusual. The other case showed a 4036 s (1 h 7 min) delay in the prediction of the capsule’s small intestine entrance. In this case, the patient had previously undergone total gastrectomy, so the capsule had entered into the small intestine directly from the esophagus at 00:01:01, and then was stuck in the blind pouch for around 50 min. Except for these three unusual cases, the differences between the model’s prediction and the ground truth were within 15 min (69/72, 95.8% of the test datasets)

As shown in Figure 7 and Figure 8, the difference between the model’s prediction and the ground truth tended to be larger when predicting the entrance to the colon compared to the small intestine. After reviewing the cases with large prediction errors, we could assume that the difficult prediction of the organ transition point between the ileum and colon may be due to the poor bowel preparation and large amount of fecal materials, which gradually deteriorates as it goes to the distal ileum (Appendix C). In these cases, the clinician also had difficulty in identifying the organ transition point. They had to review the capsule images back and forth many times to clearly confirm the transition point. In the two incomplete study cases (i.e., where the capsule did not enter the colon) for which the model incorrectly predicted the capsule’s entrance into the colon, poor bowel preparation and ulcer stricture with capsule retention seem to make the model infer that the capsule had entered the colon. 

Various deep learning algorithms have been developed so far for application to WCE, but research on organ classification and capsule localization is limited. Previous studies on this subject can be categorized based on the utilization of deep learning algorithms or not. Early algorithms employed color analysis techniques such as principal component analysis and Support Vector Machine (SVM) or considered sequence information through Hidden Markov model (HMM) [19,20,21,22,28]. However, the HMM-based algorithm causes performance degradation in video with a great deal of noise and only a feature analysis of SVM-based models was performed [20,22]. Additionally, machine learning-based organ classification was primarily reliant on color information, resulting in decreased performance when food debris was present in the small intestine and colon. Several recent studies have proposed the use of deep learning for automatic organ classification showing the superior performance of the CNN- compared to the SVM-based approach [11,12,23]. Despite these advancements, current capsule endoscopy technology faces several challenges. A major issue is that machine learning-based organ classification methods primarily rely on color information. This reliance on color leads to decreased performance when obstacles such as food debris are present in the small intestine and colon. These obstacles can significantly affect the stability and accuracy of the model, making it less reliable in clinical practice.

Our proposed method analyzes multiple frames together and integrates temporal information to address the limitations of methods that rely solely on color information. Our model maintains robust performance by combining temporal data with color information, even when color information is insufficient. This approach enhances the reliability of organ classification and localization in capsule endoscopy, ensuring more stable and accurate results in clinical practice. Our group recently published a model that detects the boundaries of the stomach, small intestine, and colon through video image reading, CNN image classification, and temporal filtering, which showed promising results [12]. To the best of our knowledge, this and the current study are the only reports that evaluate the clinical applicability of the model in a real-time localization scenario during WCE examinations, which is a crucial function in clinical practice.

Our model can be applied in clinical practice in various situations. Firstly, the automatic detection and real-time alerting of a delay in the capsule’s gastric transit time will help physicians make timely interventions according to the current guidelines, thereby preventing an incomplete study [14,15]. Additionally, this model will help future AI algorithms more accurately define abnormal findings in the gastrointestinal tract by enabling more focused analysis. Furthermore, this model can serve as the basis for the full automation of the capsule endoscopy reading process. Finally, for recently proposed methods to improve the image quality of capsule endoscopy (e.g., the diving method), having information on the moment of the capsule’s transition from the stomach to the small intestine can be useful [18].

However, the current model presents several limitations. In cases of capsule stasis in the duodenal bulb, many pyloric images can result in the model incorrectly inferring the capsule’s location to be in the stomach. This error decreases the sensitivity of the model in timely predicting the capsule’s entrance into the small intestine but does not compromise its specificity. In a clinical setting, specificity is a more critical factor in preventing incomplete studies. Secondly, the model demonstrated a substantial error in predicting the capsule’s entrance into the colon, particularly in cases of a poor bowel preparation of the small intestine. In such cases, even experts face challenges in accurately determining the transition point between the small intestine and colon. In our model, it is not possible to go through multiple images around the transition point back and forth, which is essential in this context. In addition, due to the adaptive frame rate function of the PillCam^TM^ SB3 capsule (at a range of 2 to 6 frames per second), a large time discrepancy between the model’s prediction and ground truth is inevitable when the capsule remains stationary for an extended period, such as in the cecum. Thirdly, we used previously collected capsule endoscopy data to evaluate the accuracy of the algorithm. Despite the algorithm demonstrating good accuracy and clinical usefulness when applied to real capsule video data, we did not perform a prospective study in clinical practice. Therefore, we need to assess the technical feasibility of real-time clinical application and the interaction of this algorithm with human endoscopists through a prospective clinical study. Finally, the model was trained and tested for accuracy and clinical feasibility using images from the PillCam^TM^ SB3 (Medtronic, Minneapolis, MN, USA) only, and its applicability to images from other companies remains unknown.

Based on these findings, we propose the following improvements. Increasing the diversity and complexity of the training dataset by including more cases with significant obstructions, varied anatomical structures, and abnormal transit times can help improve the model’s robustness and accuracy. Adding an anomaly detection module to the model can help identify and handle outliers and rare cases more effectively. This module would flag unusual cases that deviate significantly from the normal patterns, allowing the model to differentiate and manage these anomalies better. These proposed improvements will help address the limitations identified in our analysis and enhance the overall performance and accuracy of the model in handling challenging cases.

## 5. Conclusions

In conclusion, this study presents a real-time capsule localization model that utilizes CNN and LSTM, which demonstrated good accuracy and clinical feasibility. The algorithm showed acceptable error compared to the specialist’s label except for a few exceptional cases. We expect that our algorithm will be useful in various clinical situations. Further research is needed to assess the algorithm’s technical feasibility and its interaction with human endoscopists through prospective clinical studies. Additionally, testing the algorithm with videos from other companies and institutions is needed to demonstrate its generalizability.

## Figures and Tables

**Table 1:** The number of videos according to the underlying pathologic conditions.

Type of Main Pathology	Number of Cases
Normal	46
Bleeding lesions	20
Inflammatory lesions	30
Vascular lesions	15
Polypoid lesions	15
Total	126

**Table 2:** The number of split datasets.

Data Split	Videos	Images
Training	44	773,937
Validation	10	186,558
Test	72	1,431,967
Total	126	2,392,462

**Table 3:** The number of images for each label.

Annotation	Training Images	Validation Images	Test Images
Stomach	73,501	16,819	141,328
Small intestine	579,830	134,095	1,054,462
Colon	120,606	35,644	236,177

**Table 4:** The performance of deep learning models.

	Class	Sensitivity	Specificity	Accuracy	Error Rate	MCC	G-Mean
Without sequence awareness (baseline)	Stomach	0.949	0.991	0.987	0.013	0.927	0.970
	Small intestine	0.978	0.921	0.963	0.037	0.904	0.949
	Colon	0.890	0.987	0.971	0.029	0.893	0.937
With sequence awareness (proposed)	Stomach	0.973	0.988	0.987	0.013	0.928	0.980
	Small intestine	0.973	0.976	0.974	0.026	0.934	0.974
	Colon	0.960	0.986	0.981	0.019	0.936	0.973

**Figure A1:**
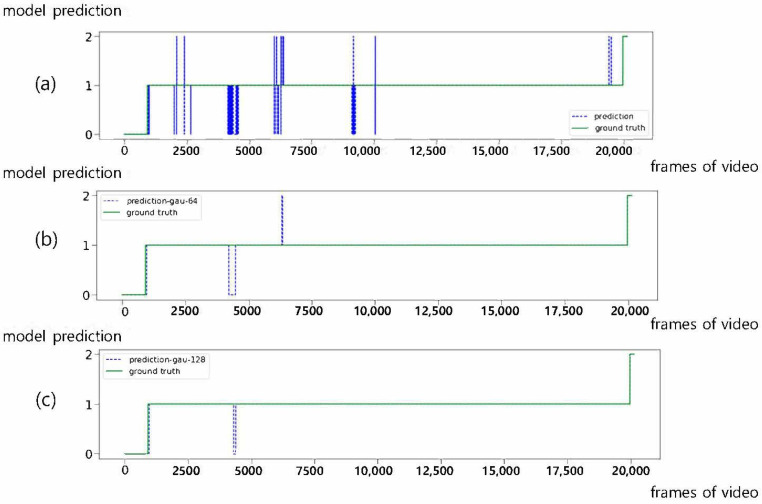
Effects of the calibration. Three graphs show the prediction results of the original model (**a**), the model with the class probability correction by applying a Gaussian filter of size 64 (**b**), and the model with the class probability correction by applying a Gaussian filter of size 128 (**c**). The X-axis represents the frames of the video. The Y-axis represents the model’s prediction, which classifies the location into the stomach (0), small intestine (1), and colon (2).

**Figure A2:**
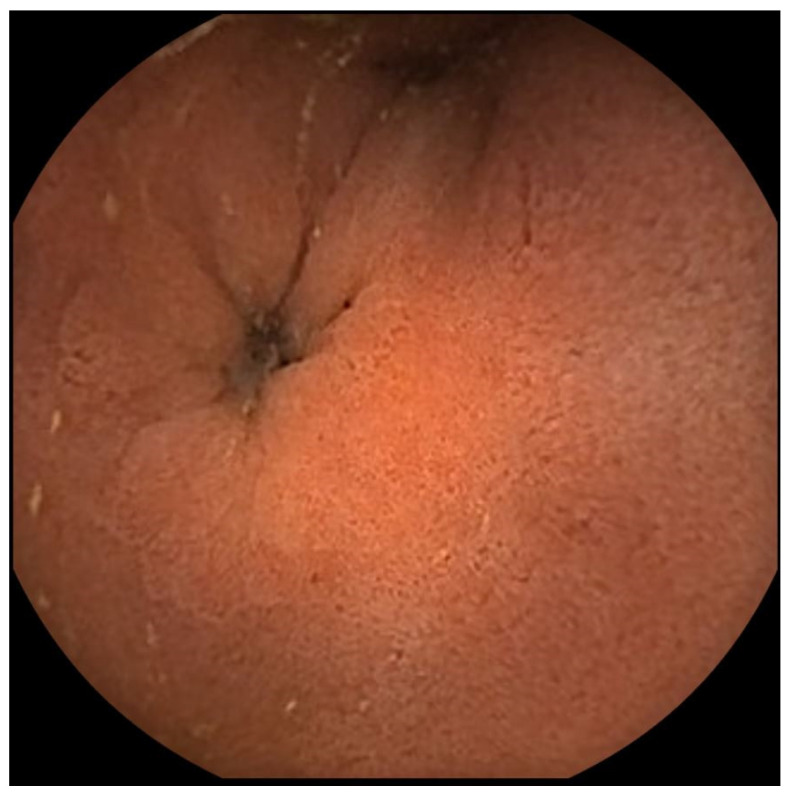
Normal pylorus image from the duodenal side.

**Figure A3:**
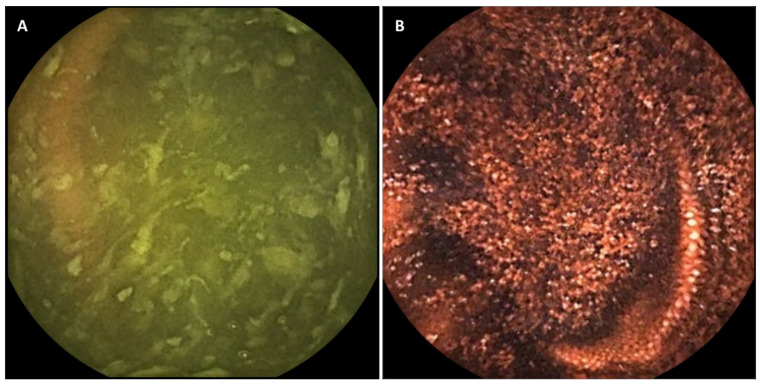
Poor bowel preparation of small intestine due to fecal material (**A**) or blood clot (**B**).

## Data Availability

Data available on request due to restrictions (privacy, and ethical reasons).

## Abbreviations

WCE	Wireless Capsule Endoscopy
CNN	Convolutional Neural Network
LSTM	Long Short-Term Memory
